# Clinical Implications of Psychophysical Olfactory Testing: Assessment, Diagnosis, and Treatment Outcome

**DOI:** 10.3389/fnins.2021.646956

**Published:** 2021-03-18

**Authors:** Baihan Su, Benjamin Bleier, Yongxiang Wei, Dawei Wu

**Affiliations:** ^1^Department of Otolaryngology, Smell and Taste Center, Beijing Anzhen Hospital, Capital Medical University, Beijing, China; ^2^Department of Otolaryngology, Massachusetts Eye and Ear Infirmary, Harvard Medical School, Boston, MA, United States; ^3^Department of Otorhinolaryngology Head and Neck Surgery, Capital Institute of Pediatrics, Beijing, China

**Keywords:** olfactory dysfunction, olfactory testing, TDI, olfactory processing, assessment, diagnosis, olfactory subcomponents, clinical relevance

## Abstract

**Purpose of Review:**

Olfactory dysfunction dramatically impairs quality of life with a prevalence of 20% in the general adult population. Psychophysical olfactory testing has been widely used to evaluate the ability to smell due to its validated utility and feasibility in clinic. This review summarizes the current literature regarding psychophysical olfactory testing and the clinical relevance of the olfactory testing with different components. Furthermore, the review highlights the diagnosis and treatment value of olfactory subtests in patients with olfactory dysfunction.

**Recent Findings:**

With the accumulation of studies of psychophysical olfactory testing in olfactory disorders, the clinical relevance of olfactory testing with different components is expanding. Different olfactory domains present with distinct olfactory processing and cortical activity. Psychophysical assessment of olfaction with three domains reveals different levels of olfactory processing and might assist with analyzing the pathophysiologic mechanism of the various olfactory disorders. Furthermore, olfactory thresholds provided the largest amount of non-redundant information to the olfactory diagnosis. Sinonasal olfactory dysfunction and non-sinonasal-related olfactory dysfunction are emerging classifications of smell disorders with certain characteristics of olfactory impairment and different responses to the therapy including steroids, sinus surgery, and olfactory training.

**Summary:**

These recent advancements should promote the understanding of psychophysical olfactory testing, the association between individual subcomponents and neurophysiological processes, and pave the way for precision assessment and treatment of the olfactory dysfunction.

## Background

Smell is one of the five basic sense which helps us understand and perceive the environment. Olfactory dysfunction dramatically impairs the quality of life with a prevalence of 20% in the general adult population (Whitcroft and Hummel, 2019). It has been shown that malnutrition, depression, increased mortality, and neurodegenerative diseases are highly associated with an impaired sense of smell (Smoliner et al., 2013; Kohli et al., 2016b; Doty, 2017; Adams et al., 2018; Liu et al., 2019). A series of etiologies lead to decreased sense of smell and approximately 200 different causes for olfactory dysfunction have been identified (Hummel et al., 2017). Olfactory dysfunction secondary to sinonasal diseases, virus infection, and head trauma account for two-thirds of patients seeking consultation from specialized smell and taste outpatient clinics. The remainder includes idiopathic, neurological-neurodegenerative, congenital, and other rare causes (Ciofalo et al., 2006; Fark and Hummel, 2013). Psychophysical olfactory testing has been widely used to evaluate the ability to smell due to its validated utility and feasibility in clinics (Doty, 2006; Rombaux et al., 2009a, c).

Furthermore, quantitative analyses with varied components facilitate assessment of the etiology, severity, treatment response, prognosis, and outcome of the olfactory dysfunction (Reden et al., 2007; Hedner et al., 2010; Devanand et al., 2015b; Whitcroft et al., 2017, 2018a; Xu et al., 2020). This review aims to summarize the current literature regarding psychophysical olfactory testing and the clinical relevance of olfactory testing with different components. These recent advancements help to promote the understanding of psychophysical olfactory testing, the association between individual subcomponents and neurophysiological processes, and facilitate the precise assessment of the olfactory dysfunction.

## Assessment of Psychophysical Olfaction

**FIGURE 1:**
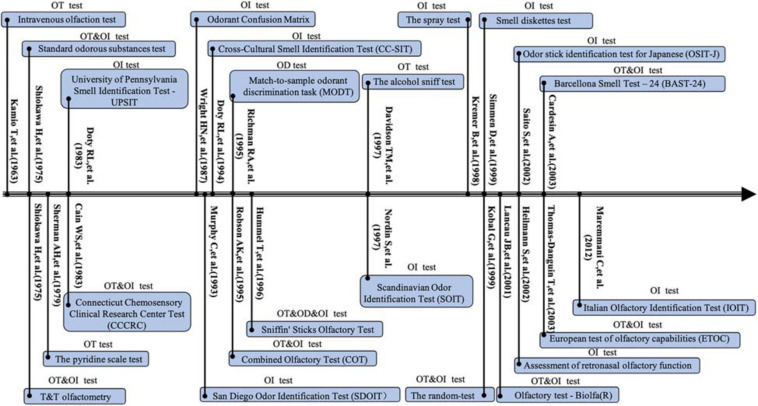
Timeline for development of the orthonasal olfaction tests between 1963 and 2012. OT, olfactory threshold; OD, olfactory differentiation; OI, olfactory identification.

**FIGURE 2:**
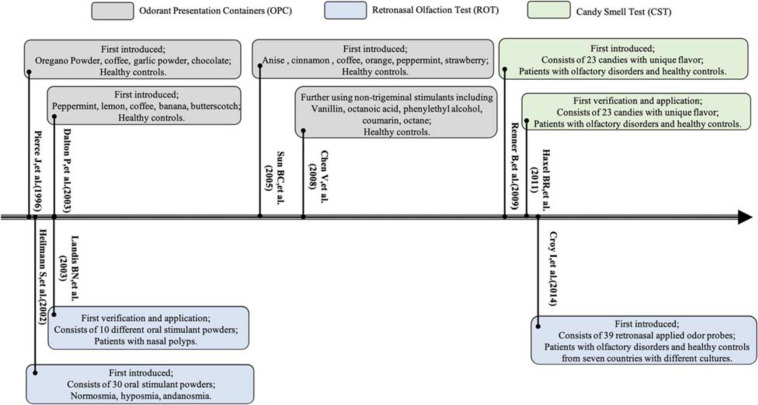
Timeline for development of the retronasal olfaction tests between 1996 and 2014.

Odors are perceived through two classic pathways: via the nostrils during sniffing, referred to as orthonasal olfaction, and via the mouth during eating and drinking, referred to as retronasal olfaction (Bojanowski and Hummel, 2012; Goldberg et al., 2018). A series of psychophysical olfactory tests have been developed to quantify the degree of olfactory dysfunction (Figures 1, 2). In general, three domains of olfaction including odor threshold, discrimination, and identification (TDI) are recognized. Individual or combinations of these olfactory subcomponents are employed to evaluate olfactory function within different types of olfactory disorders within both research studies and clinical settings. Olfactory impairment with varied etiologies involves different neuronal processes and networks. According to the location of presumed pathology, classifications of olfactory dysfunction have been proposed including conductive, sensorineural, peripheral, and central dysfunction (Hummel et al., 2017). The definition of olfactory dysfunction is based on the rough anatomical location of the lesion (Table 1). However, current psychophysical testing of orthonasal olfaction is not designed based on specific anatomical and functional distinctions in the olfactory system and the boundary between these three subcomponents remains ill-defined.

**TABLE 1:** Definition of olfactory dysfunction according to anatomical location of lesion.

Classification of olfactory dysfunction	Anatomical location of lesion
Peripheral dysfunction	Resulting from damage/loss of the olfactory processing pathways of peripheral nervous system.
Conductive dysfunction	Results from blockage of odor transmission to the olfactory neuroepithelium.
Sensorineural dysfunction	Resulting from damage/loss of the olfactory neuroepithelium or nerve.
Central dysfunction	Resulting from damage/loss of the olfactory processing pathways of the central nervous system.

## Different Olfactory Domains and Its Association With Olfactory Processing

The anatomic basis and functional requirement of olfactory subcomponents are summarized in Table 2. The methods of the olfactory testing below were standardized and widely utilized in the clinic and research centers around the world. They were recommended in the position paper on olfactory dysfunction (Hummel et al., 2017). Odor threshold (also known as sensitivity) is the lowest perceived concentration of an odorant however participants are not asked to recall or name the odorant during the test. Odor threshold for n-butanol (BUT) or phenethyl alcohol (PEA) is diluted in a solvent according to decreasing concentrations (usually two-fold dilution or three-fold dilution). It is assessed using a single-staircase procedure with three alternative choices. Here we take Sniffin’ Sticks olfactory test as an example (Hummel et al., 1997). This test comprised 16 triplets of pens with numbers from one to sixteen with pen No. 1 being the most intensely odorant with the highest concentration of BUT/PEA. The test usually starts with pen No. 16. Only one of the three pens contains an odor while the other two pens are odorless. Subjects are required to correctly detect the odor-containing pen. This test is thought to be a more direct indicator of the primary level of olfactory processing which occurs within the peripheral olfactory system. Odor threshold testing is often utilized to distinguish peripheral olfactory dysfunction at the intranasal receptor level from impairment of the central olfactory system. It is important to point out that the “threshold test” is not limited to the “intensity threshold” at which the subject can smell the smell, but also includes the shortest time that the subject can detect the odor, that is, the “time threshold” (Kamio, 1963). In addition, the purpose of the alcohol sniff test is to detect the farthest distance the subject can detect the smell, that is, the “spatial threshold” (Davidson and Murphy, 1997). However, most of the current olfactory threshold tests are aimed at the odor intensity threshold and another two threshold tests including time threshold and spatial threshold should be further explored and utilized in the clinic.

**TABLE 2:** Anatomic basis and functional requirement of olfactory subcomponents.

Subcomponents	Definition	Anatomic basis and functional requirement	Distinct patterns of cortex activity
Odor threshold	The lowest perceived concentration of an odorant	Peripheral olfactory processing in the epithelium	Bilateral piriform and orbitofrontal regions
Odor discrimination	Detect similarity and differences between odorants	Central olfactory processing including short-term working memory and executive function	Hippocampus
Odor identification	Identify an odor correctly	Olfactory detection ability and semantic memory	Broca’s area and the left inferior frontal lobe

Odor discrimination is a form of suprathreshold test that assesses the participant’s ability to discriminate between stimuli of different quality (Hummel et al., 1997). The odor discrimination test involves triplets of pens, two with the same scent and one with a different scent. Subjects are required to identify which pen contains the different scent. This test is comprised of 16 triplets of pens with numbers from one to sixteen. Similar to odor threshold tests, discrimination tests do not identify the odor. However, the participant is required to detect similarities and differences between odorants. Short-term working memory and executive function are required for decision-making in the odor discrimination test. Discrimination by quality is more cognitively loaded than intensity discrimination due to a wider activation of the cortical network.

Odor identification is another common suprathreshold test that assesses the participant’s ability to correctly identify an odor, usually through the use of verbal or visual cues (Doty et al., 1984; Hummel et al., 1997). Odor identification consists of 16 common odors that are presented individually with four given options (Hummel et al., 1997). The options are generally accompanied by pictures to reduce the bias caused by different ethnic cultural backgrounds and educational levels (Richman et al., 1995; Kobayashi et al., 2006; Krantz et al., 2009). When olfactory identification test was conducted among children, cards showing pictures-related odors were helpful (Ottaviano et al., 2018a). Odor identification task relies on both olfactory detection ability and semantic memory (Devanand et al., 2010). A patent nasal airway, appropriate sensory activation at the neuroepithelium level, and higher brain function for the perception and translation of that information to semantic verbal labels are essential for olfactory identification (Xydakis and Belluscio, 2017).

The retronasal olfactory test is a special type of olfactory identification test. Retronasal olfaction is the major contributor to flavor perception. It is supported by taste perception, i.e., sweet, salty, sour, bitter, and umami, and the perception of the texture, temperature, etc. (Goldberg et al., 2018). The orthonasal olfactory test requires the subject to sniff the odor through the anterior nostril. During the retronasal olfactory test, a powdered odorant is put on the middle of the subject’s tongue, then the subject retracts the tongue, closes the mouth and exhales through the posterior nostril (Heilmann et al., 2002). After the odor in the mouth enters the nasal cavity with the exhaled airflow through the posterior nostril, a sense of smell is generated, and the patient is asked to choose the smell from four given options. Unlike the orthonasal route of odor presentation, the retronasal route involves aromatic compounds from chewing food within the oral cavity ascending through the oropharyngeal pathway to the olfactory mucosa. Studies have shown that orthonasal and retronasal odorant perceptions activate different neural responses in the brain’s diverse areas. The anterior cingulate cortex, the orbitofrontal cortex (OFC), and the dorsal and ventral insula show a superadditive response and become more active when a taste is perceived simultaneously with a retronasally presented odor (Small et al., 2004). Conversely, these same regions show significant deactivation when a taste is perceived simultaneously with an orthonasally presented odor. The anterior cingulate cortex, OFC, and insula have been identified as key components underlying flavor perception. Patients with olfactory disorders usually complain of loss of “taste” (Hunt et al., 2019). In fact, the retronasal olfaction and the sense of taste felt by the taste buds together constitute humans’ perception of food flavor. Therefore, the use of a retronasal olfaction test to evaluate olfactory function is of special significance.

## Functional Anatomy of Human Odor Sensation, Discrimination, and Identification

A positron emission tomography study concluded that different olfactory domains engage distinct patterns of cortical activity (Kareken et al., 2003). Sensory stimulation engages bilateral piriform and orbitofrontal regions. Discrimination involves the hippocampus, implicating its role in serial odor comparisons (olfactory working memory). Odor identification involves Broca’s area and the left inferior frontal lobe, which may reflect a combination of subvocal articulation and semantic associations. The piriform cortex (PC) is recognized as the primary olfactory cortical network (OCN) where the first cortical processing of olfactory stimuli takes place (Brunjes and Osterberg, 2015). The PC consists of two different sections-the posterior and anterior PC (Gottfried, 2010). An event-related functional magnetic resonance imaging (fMRI) study showed that pairwise odorant similarities in anterior PC activity correlated with pairwise odorant similarities in chemical properties (Fournel et al., 2016). Additionally, the posterior PC activity revealed the olfactory perceptual properties. This study provides new evidence that the extraction of physical and olfactory features was based on specific fine processing of similarities between odorous stimuli in a distributed manner within the olfactory system.

The OFC has been proven to be the main neocortical target of the primary olfactory cortex, which is one of the most well-described areas of the secondary olfactory cortex. The OFC anatomically receives nerve projections from the PC and signal transduction from the olfactory bulb (Fjaeldstad et al., 2017). Whole-brain analyses revealed a significant positive correlation of gray matter volume and olfactory function scores in the right orbital sulcus, suggesting an essential role of regional gray matter volume in the right OFC and olfactory bulb volume for olfactory performance in healthy individuals (Seubert et al., 2013). Furthermore, OFC is considered as the senior cortex for olfactory identification and mediates the conscious perception of odor. A fMRI study by Kjelvik et al. (2012) demonstrated that the correct odor identification gave rise to increased activity in the left entorhinal cortex and the OFC at the whole-brain level, and both identified and non-identified odors gave rise to an increased blood oxygenated level-dependent signal in orbitofrontal and piriform cortices. However, the response to identified odors was significantly greater than that for non-identified odors. In other words, the OFC plays a vital role in olfactory identification. However, odor identification is only a manifestation of olfaction, which requires the joint participation and coordination of most brain regions, including the hippocampal and entorhinal cortex.

According to a diffusion tensor imaging study, the hippocampus is considered as a particularly important region for the functional olfactory cortex network, which is thought to play a significant role in the progression of olfactory learning and memory (Fjaeldstad et al., 2017). One study showed that the volume of the right hippocampus demonstrated a small but significant correlation with odor threshold (Smitka et al., 2012). Similarly, an animal study supported that hippocampal involvement in the network underlying odor-discrimination learning suggesting that cooperation between the dorsal and ventral hippocampus varies with learning progress (Martin et al., 2007). Neuroimaging studies regarding the relationship between olfaction and neurodegenerative disease found that Parkinson’s disease (PD) patients with olfactory dysfunction showed reduced activity in the hippocampus (Hummel et al., 2010). Alzheimer’s Disease (AD) patients with olfactory dysfunction who got significantly lower scores in discrimination also had lower hippocampal volumes and a more pronounced reduction in cortical thickness (Lian et al., 2019).

Cognitive decline is one of the leading clinical symptoms of neurodegenerative diseases, and it is often accompanied by olfactory dysfunction. An early study by Doty et al. (1987) demonstrated that both odor identification and odor detection problems were present in dementia due to Alzheimer’s type, and the odor identification impairment may be secondary to the odor detection problem. Reduced odor identification is associated with memory impairment, smaller volumes of the hippocampal and entorhinal cortex (Growdon et al., 2015). Another neurodegenerative disorder named mind wandering was associated with distinct regions of gray matter loss, as revealed by voxel-based morphometry, predominantly in the hippocampal (Growdon et al., 2015). In general, neurodegenerative diseases involve cortices within the brain that are associated with olfactory discrimination and identification. Such patients therefore tend to experience a decrease in olfactory discrimination and identification. This has also been demonstrated in studies in a healthy population. Previous studies showed that it was odor discrimination and identification not odor thresholds that correlated significantly with tests of executive function and semantic memory which is highly associated with the central processing and cognitive function (Nasreddine et al., 2005; Hedner et al., 2010).

## Diagnosis Value of Olfactory Subtests in Patients With Olfactory Dysfunction

Psychophysical assessment of olfaction with three domains reveals different levels of olfactory processing and might assist with analyzing the pathophysiologic mechanism of the various olfactory disorders. To quantify the degree of olfactory dysfunction, multicomponent psychophysical tests have been developed. The use of individual or pairs of subcomponents to diagnose olfactory impairment was less sensitive than using composite TDI scores (Lötsch et al., 2008). A machine-learned analysis of the diagnosis value of olfactory subtests suggested that olfactory thresholds provided the largest amount of non-redundant information to the olfactory diagnosis (Lötsch and Hummel, 2019). The position paper on olfactory dysfunction recommends that psychophysical assessment tools used in clinical and research settings should include reliable and validated tests of odor threshold, and/or one of odor identification or discrimination (Hummel et al., 2017).

## Clinically Meaningful Classification of Olfactory Dysfunction

Previous studies have indicated that olfactory impairment patterns obtained from psychophysical olfaction may provide diagnostic information of olfactory dysfunction (Jones-Gotman and Zatorre, 1988; Hornung et al., 1998; Whitcroft et al., 2017). The olfactory dysfunction classification based on the putative underlying etiology has been proposed to aid in patient counseling or further surgical intervention (Bonfils et al., 2009; Fonteyn et al., 2014; Hummel et al., 2017). The main causes of olfactory dysfunction include olfactory dysfunction secondary to sinonasal disease, post-traumatic olfactory dysfunction, post-infectious olfactory dysfunction, olfactory dysfunction associated with neurological disease, olfactory dysfunction associated with aging, olfactory dysfunction associated with exposure to drugs/toxins, idiopathic olfactory dysfunction, and other possible causes (sinonasal and skull base surgery, laryngectomy, tumors, multiple systemic co-morbidities, etc.) (Hummel et al., 2017; Fokkens et al., 2020). A consensus of a clinically meaningful classification based on clinical characteristics and treatment options has been reached among the rhinologists and related chemical sense researchers. Sinonasal olfactory dysfunction and non-sinonasal-related olfactory dysfunction are emerging classifications of smell disorders (Wolfensberger and Hummel, 2002; Fonteyn et al., 2014; Yan et al., 2019). Other classifications of olfactory dysfunction based on the clinical characteristics are listed in Table 3. This review will focus on the sinonasal and non-sinonasal-related olfactory dysfunction.

**TABLE 3:** Classifications of olfactory dysfunction.

Classification	
Putative underlying etiology	Sinonasal olfactory dysfunction
	Non-sinonasal-related olfactory dysfunction
Characteristics of symptom onset	Gradual/progressive onset
	Sudden onset and congenital
Genetics	Congenital olfactory dysfunction
	Acquired olfactory dysfunction
Location of presumed pathology	Conductive olfactory loss
	Sensorineural dysfunction
	Central dysfunction

### Sinonasal Olfactory Dysfunction

Sinonasal disease produces an obstructive or conductive olfactory loss that often responds dramatically to appropriate therapy including steroids and sinus surgery (Seiden and Duncan, 2001). It has been reported that sinonasal inflammatory diseases account for the major and common causes of gradual or progressive loss of smell (Enriquez et al., 2014). Sinonasal olfactory dysfunction especially chronic rhinosinusitis (CRS) related olfactory dysfunction has been thoroughly studied and a series of distinct clinical features have been identified (Fokkens et al., 2020; Mullol et al., 2020). Peripheral olfactory impairment, olfactory fluctuation, steroid-dependent reverse, a substantial benefit from endoscopic sinus surgery and preserved retronasal olfactory function are the main features of CRS-related olfactory dysfunction (Apter et al., 1999; Stevens, 2001; Kohli et al., 2016a; Whitcroft et al., 2017; Ganjaei et al., 2018; Othieno et al., 2018; Wu et al., 2018, 2020).

More importantly, analysis of olfactory subcomponent test score in patients with sinonasal olfactory dysfunction showed that odor threshold score was particularly impaired but odor discrimination and identification was relatively preserved (Whitcroft et al., 2017). Imaging studies showed that olfactory bulb volume in patients with CRS significantly increased after treatment (Gudziol et al., 2009; Herzallah et al., 2013; Sadeghi et al., 2015; Shehata et al., 2018; Whitcroft et al., 2018b). The increase in olfactory bulb volume significantly correlated with an increase in odor threshold but not with changes in odor discrimination or odor identification (Gudziol et al., 2009). Gray matter volume within the primary and secondary olfactory cortices was also significantly increased 3 months after surgical treatment for CRS (Güllmar et al., 2017; Whitcroft et al., 2018b). Furthermore, an animal study demonstrated that neuroplastic changes in the olfactory bulb were directly associated with nasal inflammation (Hasegawa-Ishii et al., 2019) and loss of olfactory sensory neuronal activity rather than neuroinflammation in the olfactory bulb was the major cause of inflammation-induced olfactory bulb atrophy (Hasegawa-Ishii et al., 2020). It can be inferred that controlled nasal inflammation by surgical procedure or medical treatment contributes to the improvement of peripheral olfactory function and subsequent recovery of the olfactory bulb volume and gray matter volume.

Landis et al. (2003) found that retronasal odor identification was significantly better than orthonasal odor identification in patients with nasal polyps. It may be associated with the presence of a mechanical obstruction in the anterior portion of the olfactory cleft. However, a recent study showed a strong correlation between retronasal and total orthonasal olfaction scores in patients with CRS (Othieno et al., 2018). Furthermore, patients with CRS demonstrated deficits in retronasal olfaction, with worse scores in patients with nasal polyps, asthma, and aspirin-exacerbated respiratory disease. Retronasal olfaction scores correlated with the degree of inflammation of the olfactory cleft and the olfactory cleft endoscopy scale was the only independent predictor of retronasal olfaction. Psychophysical and electrophysiological differences exist between orthonasal and retronasal olfaction, even in the absence of sinus or nasal disease (Landis et al., 2005).

Interestingly, it was the odor discrimination score not odor threshold and odor identification score that significantly increased in patients with CRS after multimodal treatment, which included endoscopic sinus surgery, oral antibiotics for 5 days, oral steroids for 12 days, and at least 6 weeks of topical nasal steroids (Walliczek-Dworschak et al., 2018). Besides, odor discrimination has been identified as the best component to reflect overall olfactory function changes during treatment for CRS (Whitcroft et al., 2018a). Distinct olfactory characteristics in terms of different olfactory domains and their association with the natural course, treatment outcome, and olfactory plasticity provide critical neurophysiological information which in turn facilitates disease-specific assessment and treatment.

### Non-sinonasal-Related Olfactory Dysfunction

Different from sinonasal olfactory dysfunction, non-sinonasal-related olfactory dysfunction is characterized by central dysfunction or sensorineural dysfunction. Non-sinonasal-related olfactory dysfunction does not present with olfactory fluctuation or steroid-dependence. Current evidence demonstrates that non-sinonasal-related olfactory dysfunction including post-infectious and post-traumatic olfactory dysfunction may not benefit from topical or oral steroids and there are limited options for non-sinonasal-related olfactory dysfunction (Yan et al., 2019). No effective treatments for non-sinonasal-related olfactory dysfunction are available at the present time except the olfactory training. It should be pointed out that olfactory training has been proven to be a potential treatment modality for olfactory dysfunction resulting from multiple etiologies including post-infectious, post-traumatic, idiopathic, and aging-related olfactory dysfunction (Sorokowska et al., 2017; Birte-Antina et al., 2018; Lamira et al., 2019; Pellegrino et al., 2019; Kattar et al., 2020). This emerging simple and effective protocol has been widely studied in patients with non-sinonasal-related olfactory dysfunction.

A study by Hummel et al. (2009) showed that patients with non-sinonasal-related olfactory dysfunction experienced a significant increase in olfactory function after receiving olfactory training, further supporting the therapeutic effect of olfactory training. Generally, patients with olfactory loss undergoing olfactory training experienced a significant increase in olfactory function with a mean improvement of 10.3 points on TDI score (Hummel et al., 2009; Pekala et al., 2016). Regardless of the varied causes of olfactory dysfunctions, a meta-analysis showed that subcomponents of olfactory function responded differently to the olfactory training. Specifically, it was the odor discrimination and odor identification but not odor thresholds that improved after olfactory training (Pekala et al., 2016). Factors associated with olfactory recovery after olfactory training have been identified in patients with olfactory dysfunction caused by various etiologies (Yan et al., 2018; Liu et al., 2020). Compared to patients with higher baseline of olfactory function, post-traumatic or idiopathic olfactory dysfunction, patients with post-infectious olfactory dysfunction were significantly associated with higher odds of relevant improvement after olfactory training (Liu et al., 2020). Additionally, a short course of the disease before receiving olfactory training was associated with olfactory improvement (Yan et al., 2018). These studies demonstrated that baseline olfactory performance, etiology of olfactory dysfunction, and a short course of disease were important factors associated with relevant improvement after olfactory training.

#### Characteristics of Olfactory Impairment in Post-infectious and Post-traumatic Olfactory Dysfunction

Patients with post-infectious and post-traumatic olfactory dysfunction were often studied together, mainly because those patients performed relatively well in both odor threshold and discrimination but poorly in odor identification (Whitcroft et al., 2017). Interestingly, most studies showed that patients with post-infectious and post-traumatic olfactory loss mainly improved on odor identification and discrimination after olfactory training (Konstantinidis et al., 2013, 2016), indicating a more central and less peripheral effect. The evidence points to agreement that olfactory training improves olfactory function in patients with post-infectious and post-traumatic olfactory loss which seems to be partly driven by top-down processes rather than bottom-up processes.

Traumatic brain injuries are the most common cause of olfactory dysfunction and up to 60% of patients with traumatic brain injury presented with olfactory dysfunction (Schofield et al., 2014; Drummond et al., 2015; Lecuyer Giguère et al., 2019). Three specific mechanisms have been proposed to describe the possible pathophysiology of post-traumatic olfactory dysfunction including sinonasal tract disruption, direct shearing or stretching of olfactory nerve fibers at the cribriform plate, and focal contusion or hemorrhage within the olfactory bulb and cortex (Marin et al., 2020). Any patient with post-traumatic olfactory dysfunction might be caused by the disruption of any or all of the above components and it is difficult to differentiate due to the lack of specific and comprehensive evaluation techniques in patients (Coelho and Costanzo, 2016; Limphaibool et al., 2020).

For patients with post-traumatic olfactory dysfunction caused by traumatic brain injury, olfactory training induced a significant, but transient effect on odor threshold (12 weeks) (Langdon et al., 2018) and the increase in odor threshold was significantly increased in the subgroup with anosmia, but not patients with hyposmia (24 weeks) (Pellegrino et al., 2019). It takes time for new neurons to send their axon to the olfactory bulb and make synaptic contact and the duration of therapy for this novel therapy varies from 12 to 56 weeks (Patel, 2017; Turner, 2020). It seems that changes of odor threshold correlated with the period of training and severity of olfactory loss in patients with post-traumatic olfactory dysfunction. However, for patients with post-infectious olfactory dysfunction after short-term olfactory training (16 weeks), olfactory function except the odor threshold improved and continue progressing after long-term olfactory training (56 weeks) (Konstantinidis et al., 2016).

After a 4-month of olfactory training, 67.8% of post-infectious and 33.2% of post-traumatic patients achieved an increase of more than 6 points in TDI (Konstantinidis et al., 2013); and the percentages of olfactory improvement in controls for post-infectious and post-traumatic patients is 33 and 13%, respectively. A recent meta-analysis concluded that patients with post-infectious olfactory dysfunctions had 2.77 higher odds of achieving a clinically important difference in TDI scores compared to controls after receiving olfactory training (Kattar et al., 2020). Apart from olfactory training, short-term systemic and/or topical steroids were recommended in patients with post-infectious olfactory dysfunctions in a recent evidence-based review (Hura et al., 2020). Although patients with post-infectious olfactory dysfunctions experienced significant improvement of olfaction, a mean increase of 4.47 in TDI scores did not reach the minimal clinically important difference of 5.5 after treatment with short-term systemic steroids (Schriever et al., 2012). Our recent meta-analysis of the effects of olfactory training on post-traumatic olfactory dysfunction showed that post-traumatic patients would achieve clinically significant results after olfactory training with a mean increase of TDI score of 4.61 (Huang et al., 2021). The different treatment response of the odor threshold between post-traumatic and post-infectious olfactory dysfunction reveals different plasticity of the peripheral olfactory function after olfactory training.

A study by Rombaux et al. (2006) showed a correlation between olfactory function and olfactory bulb volume, which was more pronounced for retronasal than for orthonasal olfactory function in patients with post-traumatic olfactory dysfunction. Furthermore, retronasal olfactory function was most affected in the patients with the most extensive cerebral damage and was least compromised in patients without such damage. This study indicated that post-traumatic olfactory loss was not only the result of the tearing of the fila olfactoria, but that it also resulted from lesions of cerebral areas related to the processing of olfactory information such as the OFC or the anterior temporal lobe. For patients with post-infectious olfactory dysfunction, there was a significant correlation between orthonasal and retronasal olfactory function (Rombaux et al., 2009b; Fonteyn et al., 2014). Although the orthonasal and retronasal olfactory pathway differed, orthonasal and retronasal olfaction were usually simultaneously impaired in patients with post-infectious and post-traumatic olfactory dysfunction. More studies are needed to explore their unique clinical significance.

#### Olfactory Dysfunction Associated With Neurodegenerative Disease and Normal Aging

The association between olfactory dysfunction and neurodegenerative disease and normal aging has been well established. The olfactory domains have different values in the disease progress of neurodegenerative disease and the outcome of normal aging. Olfactory dysfunction is one of the initial symptoms occurring years before motor symptoms and cognitive decline in neurodegenerative diseases such as AD and PD (Lafaille-Magnan et al., 2017; Marin et al., 2018). To be more specific, odor identification impairment, in particular, predicts the clinical transition from mild cognitive impairment (MCI) to AD in both clinical and community samples (Conti et al., 2013; Devanand, 2016; Roberts et al., 2016; Winchester and Martyn, 2020).

A meta-analysis concluded that olfactory identification was more profoundly impaired in patients with AD than in those with MCI (Jung et al., 2019). For cognitively intact older adults, odor identification deficits predict the incidence of amnestic MCI in 2–7 years (Graves et al., 1999; Schubert et al., 2008; Devanand et al., 2015a; Roberts et al., 2016; Jung et al., 2019; Windon et al., 2020). More importantly, impaired odor identification, particularly in the anosmic range, is independently associated with increased mortality in older adults in 4–5 years (Pinto et al., 2014; Devanand et al., 2015b; Liu et al., 2019). Compared with older adults with good olfaction, those with poor olfactory identification had a 46% higher cumulative risk (risk ratio, 1.46) for death at year 10 and a 30% higher risk (risk ratio, 1.30) at year 13. Similarly, after adjusting for confounding factors, older adults with anosmia had 3.37 times the odds of death as compared to older adults with normosmia, which was higher than independent of known leading causes of death. The following mechanisms did not account for increased mortality including nutrition, cognitive function, mental health, smoking and alcohol abuse, or frailty (Pinto et al., 2014). An odor recognition memory test was developed and the olfactory performance decreased significantly with increasing age, particularly after the age of 60 (Zucco, 2011). Furthermore, short-term memory influenced the performance of the standard discrimination and cued identification olfactory tasks especially in the elderly (Zucco et al., 2014).

In spite of the different topographical distribution of the pathological hallmarks in AD and PD, olfaction (odor identification, odor discrimination, and odor threshold) has been reported to be similarly damaged (Kovács, 2004). With regard to odor threshold, PD patients seem to be more impaired than AD patients, suggesting that PD patients could be more impaired on low-level perceptual olfactory tasks, whereas AD patients could be more strongly impaired on higher-order olfactory tasks, involving specific cognitive processes (Ottaviano et al., 2016). It has been suggested that an ideally olfactory battery to detect subclinical smell loss in PD should include olfactory identification and odor threshold. Interestingly, orthonasal and retronasal scores were not significantly correlated in PD patients and it was orthonasal not retronasal olfaction that was specifically impaired in PD patients (Ciofalo et al., 2006; Aubry-Lafontaine et al., 2020). This evidence indicates that the retronasal olfactory system was relatively unimpaired and the decrease in flavor perception is less pronounced in PD patients than what one would expect from the degree of orthonasal olfactory impairment. Further studies should evaluate both the orthonasal and retronasal olfaction in patients with the prodromal condition due to neurodegenerative disease.

#### Other Types of Non-sinonasal-Related Olfactory Dysfunction

Kallmann syndrome (KS) is a rare heterogeneous inherited disorder, characterized by hypogonadotropic hypogonadism and hyposmia or anosmia. The hypoplasia of olfactory nerve axons during the growth of the patient leads to the olfactory dysfunction (Seminara et al., 1998). A study demonstrated that KS patients presented with a reduction of olfactory bulb volume, olfactory sulcus depth and all of the three subtests of TDI scores when compared with controls (Ottaviano et al., 2015). In addition, there was a significant and positive correlation between TDI scores and total olfactory bulb volume.

Chronic renal failure (CRF) may be defined as a chronic and progressive deterioration of the metabolic and endocrine functions, resulting from impaired glomerular filtration and fluid-electrolyte imbalance. Impaired olfactory function has been found in patients with CRF (Landis et al., 2011). Frasnelli et al. (2002) reported up to 56% of patients with CRF presented with objective olfactory loss and it was readily reversible by hemodialysis or kidney transplantation (Griep et al., 1997). It has been shown that CRF affects peripheral olfactory pathways on different levels (Landis et al., 2011; Nguyen et al., 2016). A study showed that the TDI scores of the dialysis groups were significantly better when compared with no-dialysis group (Koseoglu et al., 2017). In addition, OT scores were significantly better in dialysis groups when compared with no-dialysis group, while OD or OI scores did not show any differences between two groups.

The relationship between olfaction and the number and type of drugs taken among older adults has been investigated (Ottaviano et al., 2018b). There was no association between odor identification and number of drugs taken in the elderly. But the number of drugs taken in the elderly correlated directly with a worse olfactory threshold. Volunteers taking only one drug with no influence on olfaction scored significantly better in olfactory threshold test when compared with volunteers taking five or more drugs. High consumption of calcium channel blockers, β-blockers, acetylsalicylic acid posed a negative effect on the olfactory threshold. Furthermore, the consumption of acetylsalicylic acid and potassium-sparing diuretics also correlated inversely with odor identification. It can be inferred that the number of drugs taken in elders significantly influenced the olfaction and drugs were a risk factor for olfactory impairment in the elderly.

## Conclusion

With the accumulating studies regarding psychophysical olfactory testing in olfactory disorders, the clinical relevance of the olfactory testing with different components has been increasingly supported. Different olfactory domains demonstrated specific associations with central olfactory processing within primary and secondary olfactory cortices. Psychophysical assessment of olfaction with three domains may assist with analyzing the pathophysiologic mechanism of the various olfactory disorders. Olfactory thresholds appear to provide the largest amount of non-redundant information to the olfactory diagnosis. Clinically meaningful classifications of olfactory dysfunction based on clinical characteristics and treatment options have been proposed to enhance the precision of assessment and potential treatment response. However, many open questions remain unanswered which require future investigation. First, a novel olfactory testing with more diagnostic information should be designed and developed based on the specific olfactory anatomy. Additionally, a comprehensive and joint olfactory analysis inclusive of functional imaging will likely help detect functional areas related to the olfactory processing. Moreover, research on the key mechanism of olfactory training in improving olfactory performance could provide deeper insights to the olfactory neuroplasticity.

## Author Contributions

All authors have made substantial contributions to the conception, analysis, and interpretation of data in this article, approved the submitted version, and agreed both to be personally accountable for our contributions and to ensure that questions related to the accuracy or integrity of any part of the work, even ones in which we are not personally involved, are appropriately investigated, resolved, and the resolution documented in the literature.

## Conflict of Interest

The authors declare that the research was conducted in the absence of any commercial or financial relationships that could be construed as a potential conflict of interest.

## Abbreviations

TDI: threshold, discrimination, and identification
BUT: n-butanol
PEA: phenethyl alcohol
PC: piriform cortex
OCNs: olfactory cortical networks
fMRI: functional magnetic resonance imaging
OFC: orbitofrontal cortex
CRS: chronic rhinosinusitis
AD: Alzheimer’s disease
PD: Parkinson’s disease
MCI: mild cognitive impairment
KS: Kallmann syndrome
CRF: chronic renal failure.
